# Dietary supplementation of *Scutellaria baicalensis* extract during early lactation decreases milk somatic cells and increases whole lactation milk yield in dairy cattle

**DOI:** 10.1371/journal.pone.0210744

**Published:** 2019-01-23

**Authors:** K. E. Olagaray, M. J. Brouk, L. K. Mamedova, S. E. Sivinski, H. Liu, F. Robert, E. Dupuis, M. Zachut, B. J. Bradford

**Affiliations:** 1 Department of Animal Sciences and Industry, Kansas State University, Manhattan, Kansas, United States of America; 2 CCPA Group, Janze, France; 3 Agriculture Research Organization, Volcani Center, Rishon Lezion, Israel; University of Illinois, UNITED STATES

## Abstract

Systemic inflammation is common in early lactation dairy cows and is associated with decreased milk production. The *Scutellaria baicalensis* plant contains flavonoids with anti-inflammatory and anti-oxidative properties, which may counteract the inflammatory state in early lactation dairy cows. The objective of this experiment was to determine whether *Scutellaria baicalensis* extract (**SBE**), a source of bioactive flavonoids, would alter the adaptation to lactation. Multiparous Holstein cows (*n* = 122) were used in a randomized block design to determine the effect of short-term and long-term postpartum administration of SBE on 305-d milk yield, 120-d milk component yield, and early lactation milk markers of inflammation and metabolic function. Treatments were 1) control, 2) short term (5-d) administration of the SBE (**SBE5**), and 3) long term (60-d) administration of the SBE (**SBE60**). Treatments were included in a treatment pellet that was identical to a control pellet in ingredient source and composition except for the extract (10 g/d SBE providing 3.3 g/d of the flavonoid baicalin), both provided via an automated milking system beginning on d 1 of lactation. Milk samples were collected on d 1, 3, and once during d 5–12 of lactation, followed by weekly sampling until 120 days in milk (**DIM**). Milk samples collected in the first 2 wk were used for biomarker analysis (haptoglobin, β-hydroxybutyrate [**BHB**], and glucose-6-phosphate [**G6P**]), and all samples were used for composition analysis. Cows were body condition scored every 2 wk prepartum and postpartum. Milk production, programmed pellet allocation, and actual provision of both pelleted feeds were recorded daily. Treatment effects were evaluated by contrasts between control and SBE5 and control and SBE60 for both the treatment (**TP**; wk 1–9) and carryover periods (**CP**; wk 10–37). Total pellet offered was greater for SBE60 in both the TP (*P* < 0.01) and CP (*P* = 0.02) but was not different for SBE5 during either period (*P* ≥ 0.13). No treatment effects were observed for body condition score (**BCS**), milk haptoglobin, BHB, or G6P. SBE5 did not alter milk yield or milk components. SBE60 increased whole-lactation milk yield by 1,419 kg (13%; *P* = 0.03). SBE60 increased milk lactose and fat yields (*P* ≤ 0.04) and tended to increase milk protein yield (*P* = 0.09) during TP, and each increased during CP (*P* ≤ 0.04). Somatic cell count decreased by 10% in SBE60 during TP (*P* = 0.02) but not CP (*P* = 0.13). Mastitis incidence tended to differ by treatment, being lesser for both SBE5 and SBE60 vs. control (14 and 15% vs. 33%). SBE supplementation did not impact time to pregnancy or hazard of leaving the herd. In conclusion, despite no detected treatment effects on BCS or milk biomarkers of inflammation and metabolic status, supplementation of postpartum dairy cows with *Scutellaria baicalensis* extract for 60 d was effective at increasing whole lactation milk yield.

## Introduction

Inflammation during the transition period of dairy cows has been well established and was recently reviewed [1]. Many conditions during the transition to lactation may contribute to inflammation, including metabolic disease, infectious disease, and environmental stressors [2]. Early lactation inflammation, indicated by elevated postpartum serum haptoglobin, has been associated with reduced milk yield and reproductive performance [3]. When allocated into quartiles based on week 1 plasma haptoglobin concentrations, cows in the upper two quartiles had negative shifts in energy metabolism, although cows in the middle quartiles suffered the greatest decline in dry matter intake (**DMI**), and body weight loss continued through week 9 of lactation [4]. Cows in the highest quartile for liver activity index (LAI; based largely on plasma acute phase proteins) produced 20% more milk in the first month of lactation compared to those in the lowest quartile [5].

Previous research has shown that administration of the nonsteroidal anti-inflammatory drug (**NSAID**) sodium salicylate during the first week of lactation increases whole-lactation milk and fat yield in older cows (3+ parities; [6]). Carpenter and others [7] showed similar effects of a short term administration (3-d) of sodium salicylate and another NSAID, meloxicam, on whole-lactation milk and protein yields on a commercial dairy farm. Meloxicam supplementation also improved herd retention [7]. As use of NSAID during early lactation is considered off-label drug use, plant sources of bioactive polyphenols are being investigated as alternative means of reducing inflammation.

The *Scutellaria baicalensis* plant has long been used in Chinese herbal medicine for its anti-inflammatory and antioxidant effects, with flavonoids believed to elicit these effects. The four main flavonoids identified in *Scutellaria baicalensis*—baicalein, baicalin, wogonoside, and wogonin (10.1, 5.4, 3.6, and 1.3% of dry matter [8])—are all known to have anti-inflammatory effects [9]. Baicalin pretreatment of RAW cells (a macrophage-derived cell line) decreased lipopolysaccharide (LPS) -induced nitric oxide production, increased intracellular superoxide dismutase activity, and attenuated the production of pro-inflammatory cytokines [10]. Similarly, baicalein and wogonin both attenuated LPS-stimulated nitric oxide synthase induction in macrophages [11]. Baicalein also attenuated the inflammatory response to LPS-induced mastitis in mice through decreased TL4 expression, which subsequently suppressed NF-κB and MAPK signaling and decreased mRNA abundance of pro-inflammatory cytokines (TNF-α and IL-1β) [12]. Less is known on the effects of wogonoside, but it is believed to have similar anti-oxidative effects as its aglycone wogonin [13]. Although these flavonoids have exhibited therapeutic effects when evaluated independently, there is great potential for pharmacokinetic interactions among these components that may make whole plant extracts more effective than the single components [13].

Ruminal degradation likely impacts bioavailability of these flavonoids; however, the extent of degradation of these flavonoids is unknown. Through research with the flavonoid quercetin, it is known that the glycosylated or polymeric forms of flavonoids are more bioavailable to ruminants than their aglycone (free form) counterparts [14]. Microbial cleavage of the glycoside is needed before the flavonoid aglycone can be degraded [15]. Thus, the glycosides could provide some degree of protection from complete ruminal degradation of the flavonoid. This would suggest greater ruminal degradation of baicalein and wogonin, the aglycone forms of the SBE flavonoids, than baicalin and wogonoside. Although some ruminal degradation is likely, several other flavonoids fed during the transition period, also with unknown bioavailability, have increased whole-lactation milk yield [16], increased whole-lactation energy-corrected milk (ECM) yield [17], and reduced liver damage [18].

Preliminary field trials in Europe provided evidence that early lactation supplementation of SBE increased milk production during the first two months of lactation [19]. Interestingly, these milk production effects were not seen until the second month of lactation. Conducting our study on a large commercial herd with a robotic milking system allowed us to distribute the treatments on an individual cow basis, enroll a large number of cows quickly, and track whole-lactation milk yield. In doing so we were able to use cow as the experimental unit for sufficient statistical power and evaluate the timing and persistence of any observed milk response. Facility design and cow management on this farm did not allow for blood sampling, but automatic milk sampling was present. Although plasma samples would be more sensitive to changes at the systemic level, evaluation of milk haptoglobin and BHB could provide some initial insight into how SBE might alter inflammatory status and metabolism.

The objective of this study was to determine the effect of short term (5-d) and long term (60-d) administration of *Scutellaria baicalensis* extract (SBE) after calving on milk yield and milk concentrations of haptoglobin and BHB. Secondary outcomes examined were effects of SBE on milk components, milk flavonoids, somatic cell count, reproductive outcomes, and survival in the herd. Since SBE flavonoids and NSAIDs exhibit similar anti-inflammatory properties, we expected to observe similar results to our previous NSAID trials [6,7]. Thus, we hypothesized postpartum SBE supplementation would decrease inflammation and subsequently decrease markers of inflammation in milk, increase milk yield, and improve herd retention.

## Materials and methods

### Cows and treatments

Multiparous Holstein cows (n = 122) on a commercial farm were used in a randomized block design to determine the effects of short term (5-d) and long term (60-d) postpartum administration of SBE. All work was carried out with the permission of the farm owner and all procedures approved by the Kansas State University Institutional Animal Care and Use Committee. Cows were blocked by parity (2 and 3+), calving date, and risk factors (high risk block: calving difficulty score ≥ 3 or twins [20]), then randomly assigned within block to one of three treatments. Prepartum cows were housed in pens of approximately 10 cows on a bedded straw pack. Upon calving, cows were moved into a single fresh pen where they had free access to an automated milking system (**AMS**; Astronaut A3, Lely Ltd., Maassluis, the Netherlands), but were encouraged through the AMS if their voluntary attendance was less than 3 visits that day. After 3 to 5 days, unless experiencing health issues, cows were moved to one of three free-stall barns (determined by pen counts at the time) with voluntary access to three AMS units within their pen. All cows were managed according to the standard operating procedures of the farm.

Assigned milking frequency, defined as the number of AMS visits a cow is allowed to make daily, was adjusted throughout lactation on an individual cow basis accounting for production level and stage of lactation. Targeted milking frequency was determined by an algorithm accounting for both time interval and optimum expected yield per milking. Time interval between allowed visits was based on the maximum number of daily milkings which ranged from 3.5 to 6. Milking attendance was monitored twice daily and cows identified for inadequate milking frequency (last AMS visit > 8 h before) were moved to the robot for milking.

Cows were fed a partial mixed ration (**PMR**) *ad libitum* twice daily and were provided with pelleted concentrate feed in the AMS. *S*. *baicalensis* extract blended in a calcium carbonate vehicle (AXION start SB, Groupe CCPA, Janze, France) was incorporated into the dairy’s standard robot feed formulation and pelleted. The control and treatment pelleted feeds were stored in two feed bins that independently supplied the milking robots. Treatments were 1) control (*n* = 39), 2) short term (5-d) administration after calving of the Scutellaria pellet (*n* = 43; **SBE5**), and 3) long term (60-d) administration after calving of the Scutellaria pellets (*n* = 40; **SBE60**). Treatments began within 24 h after calving. All cows received the control pellet, with the amount based on stage of lactation and milk production. Treatment cows were allocated 1.8 kg of the treatment pellet (delivering 100 g test material/d) in place of an equal amount of control pellet across all milkings for either 5 or 60 d. The test material contains 10% SBE with a calcium carbonate vehicle and therefore delivered 10 g SBE and approximately 3.3 g baicalin per d [21]. During the first 50 d of lactation, total pellet allocation was based on DIM, with cows allocated increasing pellet feed, up to 3.6 kg at 20 DIM and then to 5.4 kg at 50 DIM. From d 51 until 2 weeks prior to dry off, total pellet allocation was based on a feed table which incorporated milk production and milking frequency as factors. Cows producing < 27.2 kg milk were allocated 4.5 kg of pellet, whereas pellet allowance for cows producing between 27.2 and 65.8 kg milk ranged from a minimum of 4.5 to a maximum of 7.3 kg, with the exact amount determined according to the following equation: pellet allocation (kg/d) = 0.033 × milk yield (kg/d) + 2.524. The feeding program distributed the target amount of treatment feed across the average number of daily milkings per cow. Due to the nature of AMS, voluntary deviations from a cow’s average number of milkings resulted in slight excesses or shortfall in actual provision of pellet compared to the targeted allocation, and instances in which not all the feed allocated for that particular milking was dispensed were recorded as rest feed. Reported pellet offered is the amount of pellet that was actually distributed while in the AMS.

### Data collection and sampling procedures

The PMR, control pellets, and treatment pellets were sampled biweekly and composited by month for nutrient analysis by Dairy One Forage Laboratory (Ithaca, NY). Nutrient analyses are reported as means across the study for the PMR in Table 1 and the pelleted feeds in Table 2.

**Table 1:** Nutritional composition of the partial mixed ration (PMR).

Item	% of dry matter	SD
Ingredient		
Sorghum silage	11.84	
Corn silage	14.24	
Haylage	5.56	
Alfalfa hay	13.24	
Ground corn	11.75	
Whole cottonseed	6.01	
Wet corn gluten feed^1^ [22]	37.37	
Nutrient		
Dry matter, % as-fed	57.06	0.27
Crude protein	18.71	0.37
Acid detergent fiber	20.89	1.54
Neutral detergent fiber	31.96	2.31
NE_L_^2^, Mcal/kg	1.65	0.04

^1^OneTrak (Cargill Inc., Blair, NE) is a corn milling product that includes supplemental protein, vitamins, and minerals

^2^Estimated according to NRC (2001)

**Table 2:** Ingredient and nutritional composition of the control and treatment pellet.

Item	Control pellet	Treatment pellet	SD
Ingredient, % of dry matter			
Ground corn	42.47	42.44	
Wheat middlings	27.76	27.23	
Wheat flour	15.16	10.10	
Soybean meal (47.5%)	10.92	10.92	
Molasses	3.16	3.16	
Super bind^1^	0.53	0.53	
Test feed premix^2^	-	5.62	
Nutrient, analyzed, % of dry matter (unless otherwise specified)			
Dry matter, % as-fed	87.60	87.44	0.81
Crude protein	17.33	17.30	0.38
Acid detergent fiber	6.77	5.30	0.70
Neutral detergent fiber	15.33	14.52	0.94
NE_L_^3^, Mcal/kg	1.94	1.94	0.02

^1^Modified lignin sulfonate pellet binder (Bonaventure Chemicals, Inc., Weston, FL)

^2^Test feed premix included wheat flour, calcium carbonate, and *Scutellaria baicalensis* extract

^3^Estimated according to NRC (2001)

Milk samples were collected on d 1, 3, and once during d 5–12 of lactation, followed by weekly sampling for the remainder of the 120-d collection period, into vials containing a preservative tablet (2-bromo-2-nitropropane-1,3diol). Milk samples, collected at one milking on each of the specified days, were representative of the complete milking; the d 1 sample was collected from the first milking of the lactation (colostrum). Milk samples collected in the first 2 wk of lactation were allocated for biomarker analysis (haptoglobin, β-hydroxybutyrate [**BHB**], glucose, and glucose-6-phosphate [**G6P**]) and composition analysis; subsequent samples were used only for composition analysis. Subsampled milk was centrifuged at 2,600 × *g* for 10 min. Skim milk was transferred to microcentrifuge tubes and frozen -20°C until haptoglobin, BHB, glucose, and G6P analyses.

Cows were scored every 2 wk for body condition on a 5-point scale (1 = extremely thin to 5 = extremely obese) [23] from wk -3 to wk 17 relative to calving. Daily milk production, DIM, number of milkings per day, programmed feed daily allocated and feed provided for both pelleted feeds, and rumination data were recorded on an individual cow basis and collected using the management software, Time for Cows (**T4C**; Lely). Cows all wore neck collars containing rumination monitors (Qwes-HR, Lely) with a microphone that uses the distinctive sounds of rumination and regurgitation to generate data surrounding rumination. A reliability score (1–100%) which is based on the proportion of the day in which sound bites are recorded, was assigned to daily data. Only rumination data with ≥ 80% reliability were used for analysis. Pregnancy was determined by pregnancy-associated glycoproteins in milk samples (MQT labs, Kansas City, MO) collected between 32–38 d post-breeding and verified in samples from 74–80 d post-breeding. Pregnancy-associated glycoproteins were measured by an ELISA (IDEXX, Westbrook, ME) with pregnancy declared when optical density > 0.25. Culling data and time to pregnancy were reported in PC Dart (Dairy Records Management Services, Raleigh, NC) by the farm staff.

### Sample analyses

Milk samples were analyzed for concentrations of fat, true protein, lactose (B-2000 Infrared Analyzer; Bentley Instruments, Chaska, MN), MUN (MUN spectrophotometer, Bentley Instruments), and log10-transformed somatic cell count (SCC 500, Bentley Instruments) by MQT labs (Kansas City, MO).

Skim milk samples were analyzed for haptoglobin (ELISA kit #2410–7; Life Diagnostics, West Chester, PA). The preservative tablet was validated to have no effect on the results of the assay (mean haptoglobin concentrations with and without preservative: 1.44 vs. 1.66 ± 0.10; *P* = 0.45, n = 2). Prior to BHB analysis, skim milk samples were deproteinized. Milk samples were first alkalinized (pH > 9) with 50 μL 3 M KOH; this step was inadvertent but did not impact the analyte of interest, so we subsequently proceeded with the protocol as intended. A greater amount of sample (1000 μL vs. 500 μL) was used for d 1 samples than d 3 and 9 samples as total solids in colostrum samples decreased the amount of supernatant in subsequent steps. Samples were incubated in a water bath at 60°C for 1 h, then either 600 μL (1 DIM samples) or 300 μL (3 and 9 DIM samples) 3 M HClO_4_ was added, samples were mixed, and placed on ice for 10 min. After centrifugation at 10,000 × *g* for 10 min, 500 μL of supernatant was transferred to a separate 1.5 mL micro centrifuge tube and neutralized with 105 μL 3 M KOH. After incubation on ice for 10 min, samples were centrifuged for 10 min at 10,000 × *g*. The supernatant was transferred to a clean micro centrifuge tube and frozen until analysis by enzymatic kit (kit #H7587-58; Pointe Scientific Inc., Canton, MI).

Milk glucose and G6P concentrations were measured in skim milk samples by a fluorimetric assay applying enzymatic reactions as previously described [24, 25]. Glucose-6-phosphate was determined through enzymatic oxidation by G6P dehydrogenase using NADP+ and the total (both glucose and G6P) was determined by enzymatic oxidation by both G6P dehydrogenase and hexokinase. Diaphorase solution (**DS**) was prepared by combining 75 mg KCl, 4 μL Triton X-100 (1% on distilled, deionized water), 100 μL resazurin (4.8 mM; Sigma Aldrich, R7017-5G), 100 μL diaphorase (100 UN/mL; Sigma Aldrich, M9272), and adjusted to 10 mL with Tris/HCl buffer [100 mM (12.11 g/L, Sigma Aldrich, T1410), pH 7.6 with 10 mM MgCl_2_ (2.03g/l, Sigma Aldrich, M9272)]. The reaction mixture (**RM**; 1 mL used for G6P determination) included 10 μL NADP (75 mg/mL in water; Sigma Aldrich, N0505), 1 μL G6P dehydrogenase (200 UN, Sigma Aldrich, G2921), and 989 μL DS. The reaction mixture for both glucose and G6P (**RM-T**; 1 mL) included 10 μL ATP (100 mM in Tris/HCl buffer; Sigma Aldrich, A2383), 2 μL hexokinase (2000 UN/mL distilled, deionized water; Sigma Aldrich, H4502), and 988 μL RM. Standards (0, 10, 50, 100, 250, 500 μM) were made using either 10 mM G6P solution (G6P, Sigma Aldrich, G7879) for G6P or 10 mM glucose solution for total. For G6P determination, 100 μL of RM was added to 10 μL of skim milk or standard in a 96-well plate. On a separate plate, 100 μL of RM-T was added to 10 μL sample or standard for total glucose and G6P determination. Plates were incubated at room temperature for 30 min and read by fluorescence (540/590 nm). Free glucose concentration was determined by difference (total glucose and G6P –G6P).

#### HPLC analysis for flavonoids

Milk samples were analyzed by high performance liquid chromatography (**HPLC**) to determine milk concentrations of three flavonoids found in SBE (baicalin, baicalein, and wogonin), and to determine whether supplementation changed concentrations of these flavonoids in milk. Standards of baicalin (Y001273), baicalein (465119), and wogonin (681670) were purchased from Sigma Aldrich (Darmstadt, Germany). Serial dilutions of the standards (1, 2, 5, 10, 25, 50, and 100 μg/mL) were prepared in methanol. Skim milk samples from d 3 and d 9±4 were used from each of 5 control and 10 SBE60 cows selected at random. Solvent extraction was performed on skim milk samples according to [26], modified to exclude the acetic acid step. Briefly, 600 μL of methanol was added to 300 μL skim milk and mixed vigorously for 30 s. Samples were centrifuged at 14,000 × *g* for 5 min, then supernatants were collected and filtered through a 0.20 μm filter for analysis by HPLC.

Analysis of flavonoids was performed by HPLC according to the methods of Gao et al. [27] with some modifications. A Discovery BIO wide pore C18 column (150 mm × 4.6 mm, 5 μm pore size, Supelco 568222-U, Sigma-Aldrich, Darmstadt, Germany) with a guard column (4 mm × 3 mm, AJ0-4287, Phenomenex, Torrance, CA) was used for separation. Elution of the flavonoids was achieved using 0.1% formic acid (pH 2.7; eluent A), a 50:50 mixture of 0.2% formic acid and methanol (pH 3.07; eluent B), and methanol (eluent C). Due to instability, eluent B was freshly prepared daily. The mobile phase gradient program was 100% eluent A for 10 min, followed by 100% eluent B from 10.1 to 30 min, then eluent C from 30.1 to 40 min, followed by re-equilibration with eluent A from 40.1 to 50 min. The flow rate was 1 mL/min and sample injection volume was 20 μL. Flavonoids were detected by absorption at 270 nm (Acutect 500 UV/VIS Detector) and peak area was determined (SRI Instruments, Torrance, CA).

Retention times of baicalin, baicalein, and wogonin were 15.4, 21.7, and 28.5 min, respectively. Of the serial dilutions of standards (1, 2, 5, 10, 25, and 50μg/mL), concentrations were constantly detected from 50–2 μg/mL. Therefore, the limit of detection for this assay was approximately 2 μg/mL for each analyte.

To adjust for baseline noise near the baicalin peak, the peak area at the retention time of baicalin (14.51 to 15.63 min) was determined for blank (mobile phase) injections. Some samples, however, had peak areas that were lesser than that measured in the blank, and therefore the minimum area at this retention time was subtracted from all other samples to correct for background absorbance. Background correction was only necessary for baicalin.

Injections of standards (50 μg/mL) were used to determine the relationship between peak area and flavonoid concentration.

Fresh milk samples from 3 cows in the Kansas State University herd were used to assess the recovery of flavonoids in both whole milk and skim milk samples. Four samples were prepared and analyzed from each cow: a whole milk sample, a skim milk sample, a whole milk sample spiked with each of the flavonoids, and a skim milk sample spiked with each of the flavonoids (spike occurred prior to centrifugation to obtain skim milk). Flavonoid concentrations from the samples not spiked were subtracted from concentrations in the spiked samples to account for any flavonoids present in the baseline sample (i.e. conc. in whole milk sample with spike—conc. in whole milk sample). Recoveries for baicalin, baicalein, and wogonin were 81.64, 66.59, and 98.93% in whole milk and 83.28, 48.35, and 27.70% in skim milk, respectively.

### Statistical analysis

The amount of test material delivered between during the first 5 DIM was analyzed using the Mixed procedure of SAS (version 9.4, SAS Institute, Cary, NC) with the random effects of pen and cow and the fixed effects of treatment, DIM, month, parity, and the interactions between treatment and DIM and treatment and parity. Control cows were included in the first analysis to verify that the mean test material delivered was zero. Subsequently only the SBE5 and SBE60 cows were included in the analysis to get a more accurate variance around the means for these treatments.

Milk yield, milk composition, milking frequency, pellet offered, rumination time, and body condition score (**BCS**) data were summarized by week relative to calving for statistical analysis. Milk yield, milk composition, milking frequency, and total pellet intake were analyzed separately for the 60-d treatment period (**TP**; wk 1–9) and the carryover period (CP; wk 10–37). Statistical analysis was performed using SAS (version 9.4, SAS Institute., Cary, NC) to model the fixed effects of treatment, week, parity and two-way interactions of these variables, as well as the random effects of pen and cow. Additional variables (including their treatment interactions), were also tested and removed when they did not contribute significantly to the model (*P* > 0.10). Additional variables tested included risk block (high risk vs. low risk), BCS category (< 4 vs. ≥ 4 at calving), and month of calving. Body condition score and month of calving were not retained in any model. Values with Studentized residuals > 4 or < -4 were removed as outliers. Because there was a lesser proportion of high-risk cows (*n* = 3 per treatment) than low-risk cows, variables for which risk or its interactions remained in the model were analyzed again with risk removed from the model to generate unbiased LS treatment means and SEM; *P*-values from the full model were used for assessing treatment effects and interactions. Repeated measures within cow were modeled with autoregressive and heterogeneous autoregressive covariance structures, and the one with the least Bayesian information criterion was selected for each dependent variable. Repeated measures within cow for BCS and milk haptoglobin were modeled with spatial power covariance structures because of unequal spacing of time points.

Values for milk haptoglobin and G6P were log-transformed prior to statistical analysis; reported data were back-transformed. The statistical analysis of BHB, haptoglobin, glucose, and G6P in milk samples taken during d 5–12 of lactation were analyzed with day set at the median value of d 9. Analysis using the exact day of the third sample (d 5–12) did not reveal any treatment effect or interaction (all *P* > 0.20), so there was no impact on interpretation (results not shown).

Treatment effects were evaluated by contrasts between control and SBE60 and control and SBE5 for both the TP and CP. If an interaction with time was observed, contrasts within week were evaluated using the SLICE option of PROC MIXED. Significance was declared at *P* < 0.05 and tendencies at 0.05 ≤ *P* < 0.10.

Treatment effects for disease incidence were assessed by pairwise Fisher’s exact tests (JMP, v. 10, SAS Institute, Cary, NC). Treatment effects on both pregnancy risk and hazard of leaving the herd were assessed by Cox proportional hazards models and likelihood ratio tests (JMP, v. 10, SAS Institute, Cary, NC). Pregnancy data were censored for animals that died or left the herd prior to 250 DIM. Failures for herd retention were any cows that died or were culled, and cows surviving until 250 were censored. The proportional hazards models not only tested treatment, but also month of calving, risk block (high risk vs. low risk), pen, and BCS category (< 4 vs. ≥ 4 at calving). Variables were removed when *P* > 0.10.

## Results

### Treatment provision and total pellet offered

**Fig 1:**
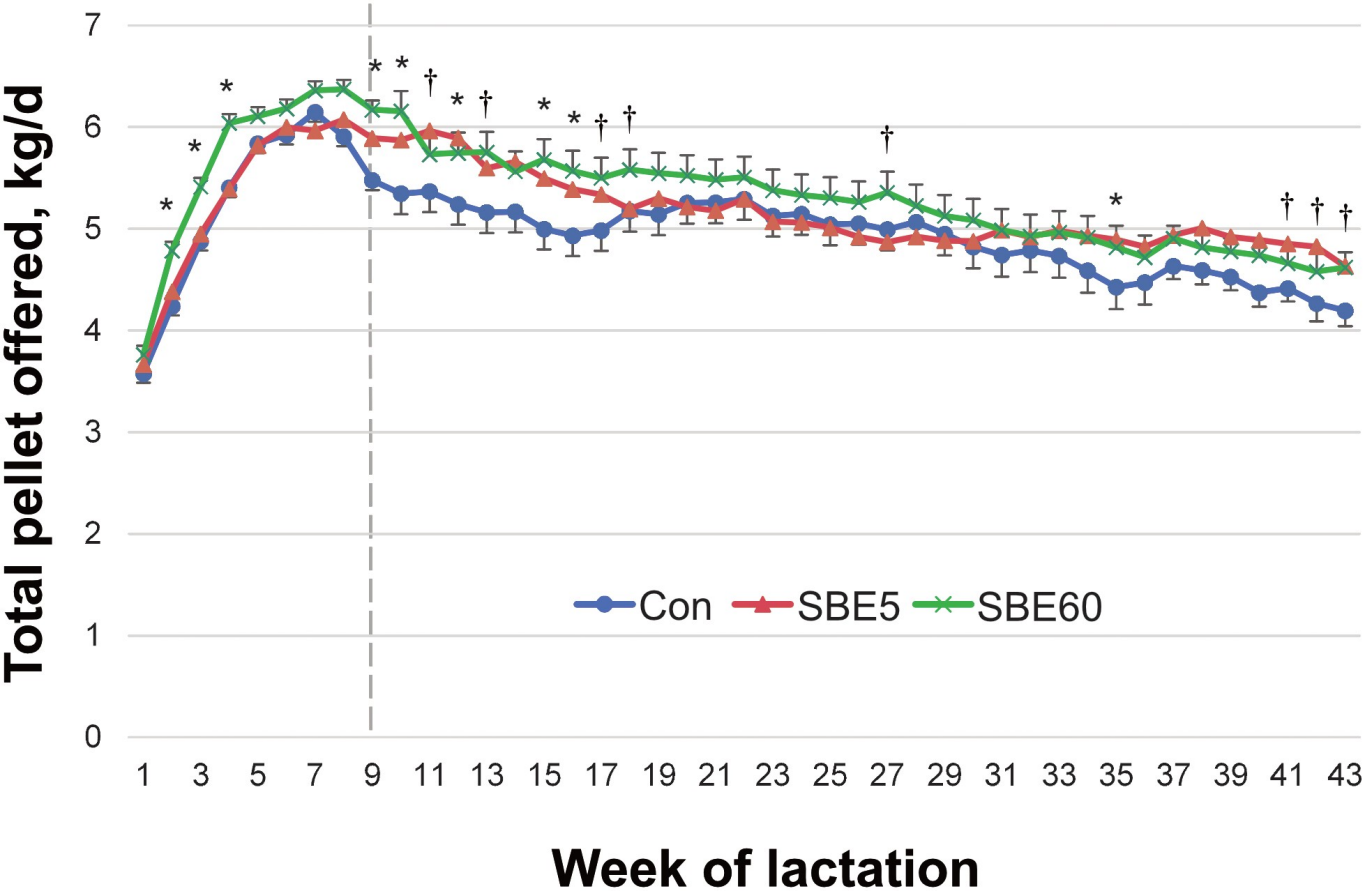
Total pellet allocated (control + treatment) to control cows and cows supplemented with *S*. *baicalensis* during the first 5 d (SBE5) or 60 d (SBE60) of lactation. Data were analyzed by treatment period (wk 1–9) and carryover period (wk 10–36). Total pellet allocation was greater for SBE60 compared to control during the wk 1–9 (*P* < 0.01) and from wk 10–43 (*P* = 0.02). Total pellet allocation did not differ between SBE5 and control during either wk 1–9 (*P* = 0.77) or wk 10–43 (*P* = 0.13). A treatment × week interaction was detected (*P* < 0.001), and differences between SBE60 and control are indicated by *(*P* < 0.05) and † (*P* < 0.10). Values are LS means ± pooled SEM, n = 39–42.

Test material delivered for the first 5 DIM was not different between SBE5 and SBE60 (*P* = 0.41; 80.8 and 83.1 ± 0.3 g/d, respectively). Test material delivered for SBE5 and SBE60 increased until reaching target amounts on day 3 as cows adapted to the AMS (mean intake of 42, 77, 102, 97, and 91 ± 4.8 g/d on d 1–5, respectively). There was an effect of DIM (*P* < 0.001) as cows adapted to the AMS; however, there was no treatment × DIM interaction (*P* = 0.94). Mean test material provision for SBE60 ranged between 92.2 and 97.8 g/d during wk 1–9 of lactation, close to the target of 100 g/d. Pellet feeding records (T4C) confirmed that no treatment feed was allocated to control cows nor to SBE5 cows after d 5 of lactation. Pellet offered was greater for SBE60 cows compared to control cows during wk 1–9 (*P* > 0.01) and wk 10–43 (*P* = 0.02). Pellet offered did not differ between SBE5 and control during either the TP or CP (both *P* > 0.10). Total pellet offered over 301 d differed by treatment (*P* = 0.03) and week (*P* < 0.001), and had a treatment × week interaction (all *P* < 0.001; Fig 1) with greater amounts of pellet offered to SBE60 than control in wk 2–4, 10–13, 15, 16, and a tendency for a difference in wk 17. Daily rumination time through 120 DIM was not different for control cows compared to either SBE5 or SBE60 in wk 1–9 or wk 10–17 (all *P* > 0.55) and no treatment × week interaction was observed (*P* = 0.39; Table 3).

**Table 3:** Weekly total pellet offered, milk yield, and milking frequency for cows fed control or *S*. *baicalensis* extract for either 5 d (SBE5) or 60 d (SBE60) following calving. Data were analyzed by treatment period (1–63 DIM) and carryover period (64–301 DIM). Values are LS means ± pooled SEM, n = 39–42.

	Control	SBE5	SBE60	SEM	*P*-values	
					Con v. SBE5	Con v. SBE60
Total pellet offered, kg/d						
d 1–63	5.25	5.30	5.66	0.14	0.77	< 0.01
d 64–301	4.97	5.18	5.28	0.15	0.13	0.02
Milk yield, kg/d						
d 1–63	42.46	44.95	47.19	2.01	0.35	0.07
d 64–301	35.39	36.23	40.02	1.91	0.73	0.04
Milking frequency, d^-1^						
d 1–63	3.24	3.34	3.48	0.21	0.60	0.19
d 64–301	2.56	2.67	2.84	0.18	0.48	0.04
Milk per visit, kg						
d 1–63	13.88	14.13	14.12	0.75	0.70	0.75
d 64–301	14.05	13.89	14.07	0.63	0.92	0.99

### Milk production and composition

**Fig 2:**
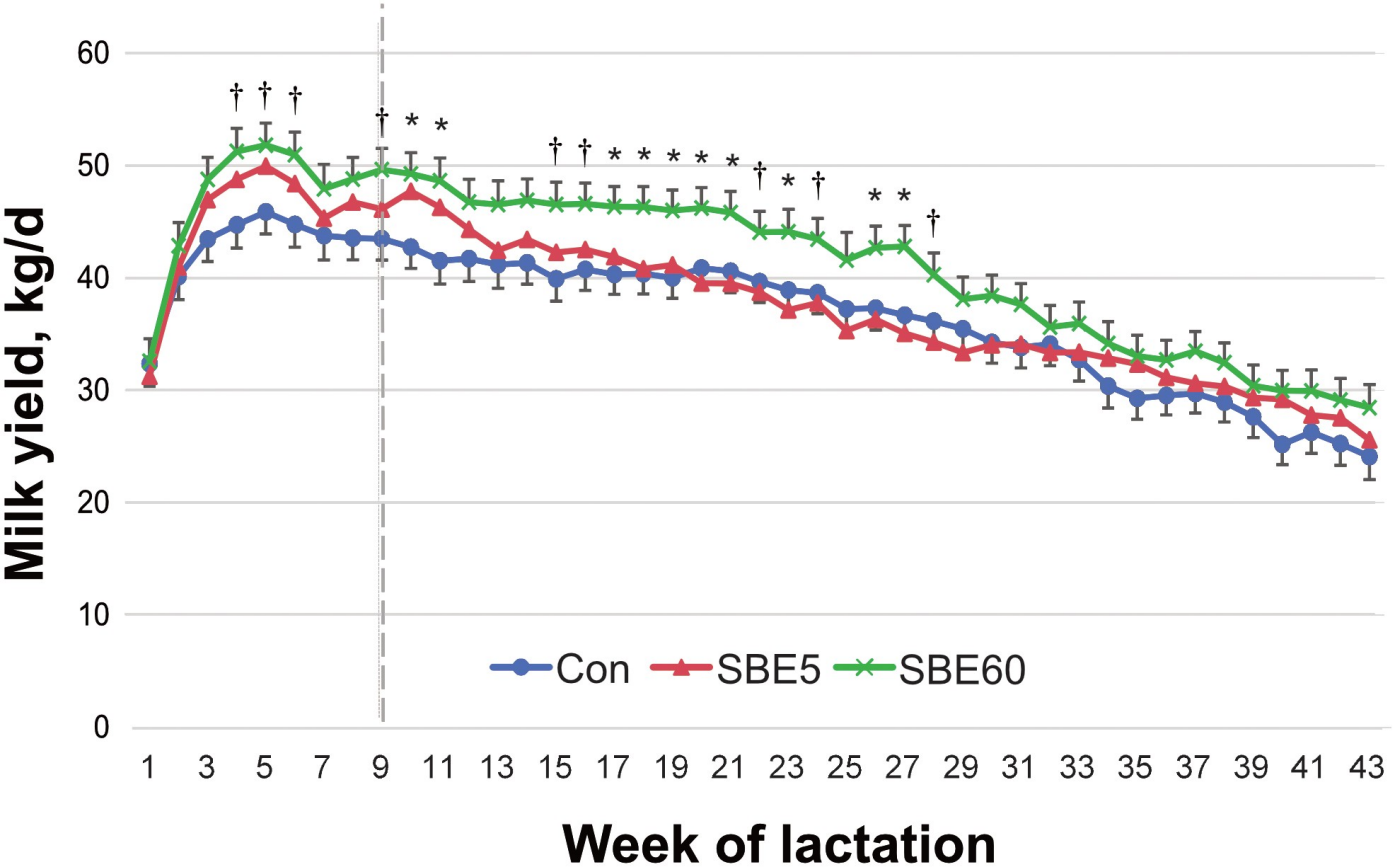
Milk yield of control cows and cows supplemented with *S*. *baicalensis* extract (SBE) during the first 5 d (SBE5) or 60 d (SBE60) of lactation. Data were analyzed by treatment period (wk 1–9) and carryover period (wk 10–43). Milk yield tended to be greater for SBE60 compared to control from wk 1–9 (*P* = 0.07) and was significantly greater from wk 10–43 (*P* = 0.04). Milk yield did not differ between SBE5 and control during wk 1–9 (*P* = 0.35) or wk 10–43 (*P* = 0.73). A treatment × week interaction was detected (*P* < 0.03), and differences between SBE60 and control are indicated by *(*P* < 0.05) and † (*P* < 0.10). Values are LS means ± pooled SEM, n = 39–42.

Milk yield did not differ between SBE5 and control either during wk 1–9 (*P* = 0.35) or wk 10–43 (*P* = 0.73). Milk yield tended to be greater for SBE60 compared to control during wk 1–9 (*P* = 0.07) and was significantly greater during wk 10–43 (*P* = 0.04). An overall treatment × week interaction was observed with tendencies for differences during wk 4–6, 9, 15–16, 22, 24, and 28 and significant differences in wk 10–11, 17–21, 23, and 26–27 (Fig 2). Whole-lactation milk yield (305-d) was 11,245, 11,608, and 12,664 ± 465.3 kg for control, SBE5, and SBE60, with significant differences between SBE60 and control (*P* = 0.03), but not between SBE5 and control (*P* = 0.60). The whole-lactation milk yield was also analyzed using milk PTA as a covariate for the 105 cows for which that was available. The results showed the same mean separation between treatments at 11,392, 112,790, and 11,131 ± 554 kg for control, SBE5, and SBE60 with differences between SBE60 and control (*P* = 0.03), but not between SBE5 and control (*P* = 0.73). Milking frequency was not affected by either SBE5 (*P* = 0.60) or SBE60 (*P* = 0.19) during the first 63 DIM, but milking frequency increased for SBE60 during the CP compared to control (*P* = 0.04) whereas no difference was detected between SBE5 and control (*P* = 0.48). As expected, milking frequency differed by week (*P* < 0.001), but no overall treatment × week interaction was observed (*P* = 0.11). Despite the difference in milking frequency, milk yield per milking did not differ by treatment during the treatment or carryover periods (all *P* > 0.65).

Milk composition data during the first 17 wk of lactation are summarized in Table 4. There were no treatment effects on milk fat or protein content during the treatment or carryover periods (all *P* ≥ 0.15). Milk lactose concentration tended to be increased for SBE60 compared to control during the TP (*P* = 0.06), but not the CP (*P* = 0.25), and did not differ for SBE5 compared to control during either the TP or CP (*P* = 0.54 and 0.46, respectively). Milk fat yield was increased by SBE60 during both the TP and CP compared to control (both *P* = 0.04), whereas SBE5 did not differ from control in either period (both *P* ≥ 0.50). Milk protein yield tended to be increased for SBE60 compared to control in the TP (*P* = 0.09) and increased during the CP (*P* = 0.01), but again did not differ between SBE5 and control (*P* ≥ 0.13). Milk lactose yield increased for SBE60 but not SBE5 compared to control during the TP (*P* = 0.03 and 0.26, respectively). During the CP, milk lactose yield continued to be greater for SBE60 compared to control (*P* = 0.02), and SBE5 tended to increase milk lactose yield compared to control (*P* = 0.07). There was a tendency for an overall treatment × week interaction for milk lactose yield (*P* = 0.08) with significantly greater values for SBE60 compared to control during wk 5–6 and 8–11, and tendencies for increases during wk 4, 14, and 15. Milk lactose yield was also greater for second lactation cows compared to cows in lactation 3+ (2.31 vs. 2.15 ± 0.06 kg/d; *P* = 0.03).

**Table 4:** Rumination time through 120 DIM and milk composition for the first 17 weeks of lactation of control cows and cows supplemented with *S*. *baicalensis* extract (SBE) for either 5 d (SBE5) or 60d (SBE60) following calving. Values are LS means ± pooled SEM, n = 39–42.

	Control	SBE5	SBE60	SEM	P-values	
					Con v. SBE5	Con v. SBE60
Rumination, min/d						
d 1–63	429.9	427.3	429.0	8.20	0.76	0.92
d 64–120	410.3	405.8	409.9	7.30	0.58	0.95
Milk fat, %						
d 1–63	3.84	3.84	3.84	0.17	0.95	0.99
d 64–120	3.24	3.08	3.29	0.18	0.28	0.77
Milk protein, %						
d 1–63	3.16	3.10	3.12	0.06	0.40	0.54
d 64–120	2.97	2.89	2.97	0.05	0.15	0.99
Milk lactose, %						
d 1–63	4.87	4.89	4.95	0.04	0.54	0.06
d 64–120	4.92	4.95	4.97	0.04	0.46	0.25
Milk fat, kg/d						
d 1–63	1.61	1.67	1.77	0.08	0.50	0.04
d 64–120	1.35	1.38	1.51	0.08	0.73	0.04
Milk protein, kg/d						
d 1–63	1.34	1.40	1.46	0.06	0.42	0.09
d 64–120	1.23	1.34	1.41	0.05	0.13	0.01
Milk lactose, kg/d						
d 1–63	2.10	2.23	2.36	0.10	0.26	0.03
d 64–120	2.07	2.28	2.35	0.09	0.07	0.02
SCC, log_10_ cells/mL						
d 1–63	2.19	2.07	1.86	0.13	0.37	0.02
d 64–120	2.13	1.98	1.91	0.14	0.29	0.13

**Fig 3:**
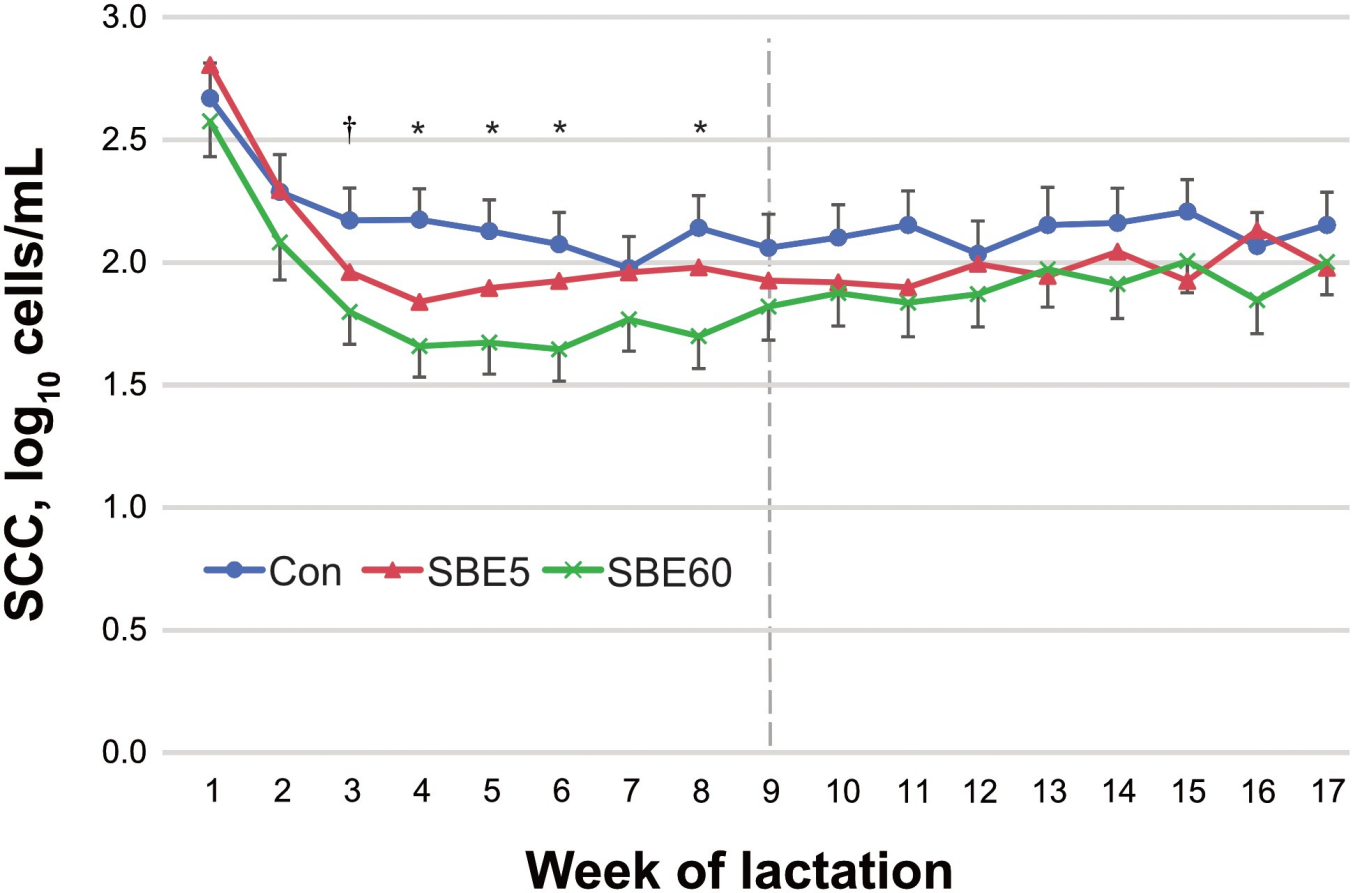
Somatic cell count (SCC) of control cows and cows supplemented with *S*. *baicalensis* extract (SBE) during the first 5 d (SBE5) or 60 d (SBE60) of lactation. Data were analyzed for the treatment period (wk 1–9) and carryover period (wk 10–17). Somatic cell count was not different between SBE5 and control during wk 1–9 (*P* = 0.37) or wk 10–17 (*P* = 0.29). Somatic cell count was decreased for SBE60 compared to control during wk 1–9 (*P* = 0.02), but not during wk 10–17 (*P* = 0.13). No treatment × week interaction was detected (*P* = 0.16). Values are LS means ± pooled SEM, n = 39–42. Differences between SBE60 and control are indicated by *(*P* < 0.05) and † (*P* < 0.10).

Somatic cell count was decreased by SBE60 compared to control during the TP (*P* = 0.02) with a tendency for a difference in wk 3 and significant effects in wk 4–6 and 8 (Fig 3). SBE5 did not affect SCC (*P* = 0.37) during wk 1–9, and neither SBE5 or SBE60 affected SCC during the CP (*P* = 0.29 and 0.13, respectively).

**Fig 4:**
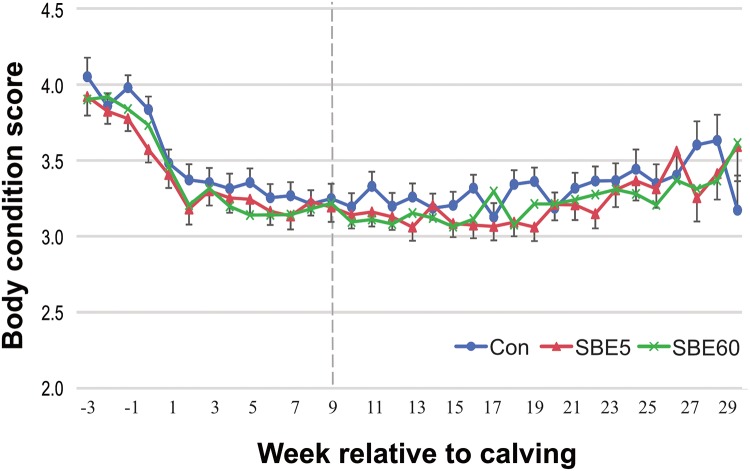
Body condition score (5 point scale) from 3 weeks prepartum to 29 weeks postpartum for control cows and cows supplemented with *S*. *baicalensis* extract (SBE) for the first 5 d (SBE5) or 60 d (SBE60) of lactation. Least square means across time were 3.40, 3.30, and 3.31 ± 0.06 for CON, SBE5, and SBE60, respectively. BCS differed by week (*P* < 0.001), but there were no differences by treatment (*P* = 0.44) or treatment × week (*P* = 0.57). Values are LS means ± pooled SEM, n = 39–42.

Overall there was no treatment effect on BCS (*P* = 0.44) with means of 3.40, 3.30, and 3.31 ± 0.06 for control, SBE5, and SBE60. As anticipated, BCS differed by week (*P* < 0.001), but there was no treatment effect on prepartum or postpartum BCS (treatment × week: *P* = 0.57). Treatment means for BCS from 3 wk prior to calving and through 29 wk of lactation are shown in Fig 4.

### Milk markers of inflammation, metabolism, and energy balance

Milk biomarkers are reported in Table 5. Neither milk haptoglobin nor milk BHB showed significant treatment effects (*P* = 0.97 and 0.89, respectively) or treatment × DIM effects (*P* = 0.45 and 0.47). Milk haptoglobin concentrations were greatest the day after calving (when inflammation is greatest) and subsequently declined for d 3 and d 5–12 milk samples (*P* < 0.001). BHB concentration also had a DIM effect (*P* < 0.0001), increasing from d 1 to d 5–12 samples.

**Table 5:** Milk haptoglobin, β-hydroxybutyrate (BHB), and glucose-6-phosphate (G6P) on days 1, 3, and 5–12 of lactation for control cows and cows receiving *S*. *baicalensis* extract (SBE) for either 5 d (SBE5) or 60 d (SBE60) after calving. Values are LS means ± pooled SEM, n = 39–42.

	Control	SBE5	SBE60	SEM	Trt	DIM	Trt×DIM
Haptoglobin, μg/mL					0.97	< 0.001	0.45
Day 1	4.98	3.54	5.47	1.04			
Day 3	1.53	1.70	1.44	0.35			
Day 5–12	0.59	0.69	0.50	0.13			
BHB, μM					0.89	< 0.001	0.47
Day 1	264.0	265.3	249.4	23.6			
Day 3	639.7	609.7	632.2	22.6			
Day 5–12	729.1	746.7	717.8	18.6			
G6P, μM					0.91	< 0.001	0.80
Day 1	914.6	884.9	889.5	56.6			
Day 3	336.1	362.7	348.8	22.5			
Day 5–12	279.8	286.4	300.6	18.5			
G6P, % of glucose					0.38	< 0.001	0.28
Day 3	78.3	78.3	85.5	0.03			
Day 5–12	64.1	63.6	64.3	0.03			

Glucose-6-phosphate measured in milk as an indicator of energy balance decreased as DIM increased (*P* < 0.001), but there was no effect of treatment (*P* = 0.91) or treatment × DIM (*P* = 0.80). Similarly, G6P expressed as a percent of glucose decreased with increasing DIM (*P* < 0.001), but did not differ by treatment (*P* = 0.38) and there was no treatment × DIM interaction (*P* = 0.28). Interestingly, milk G6P content was lesser in cows classified as high-risk (287 v. 447.6 ± 22 μM).

#### Flavonoid concentrations in milk

None of the skim milk samples had concentrations of baicalin, baicalein, or wogonin that exceeded the detection limit of 2 μg/mL before adjusting for recoveries. After the recovery adjustments, the maximum observed concentrations for baicalin, baicalein, and wogonin were 2.03, 0.39, and 2.74 μg/mL, respectively), and a baicalein peak was only detected for a single sample. Since flavonoid concentrations were below the reliable detection limit, a quantifiable concentration could not be determined in any of the milk samples.

#### Disease incidence

Incidence of disease through 250 DIM is reported in Table 6. There were no treatment effects for any diseases except mastitis (*P* > 0.10). Mastitis incidence tended to differ by treatment (*P* = 0.06), being lesser for SBE5 (*P* = 0.04) and SBE60 (*P* = 0.05) vs. control.

**Table 6:** Disease incidence through 250 DIM for control cows and cows receiving *S*. *baicalensis* extract (SBE) for either 5 d (SBE5) or 60 d (SBE60) after calving.

	Control	SBE5	SBE60
At-risk	39	43	40
Fever	3	1	1
Milk fever	1	2	2
Displaced abomasum	0	0	0
Retained placenta	2	5	4
Metritis	3	4	6
Lame	2	2	0
Off feed	3	2	1
Mastitis^1^	13	6*	6^#^
Other	0	1	2

^1^Mastitis incidence tended to differ by treatment (*P* = 0.06).

*Control vs. SBE5: *P* = 0.04

^#^Control vs. SBE60: *P* = 0.05.

#### Time to pregnancy and herd retention

**Fig 5:**
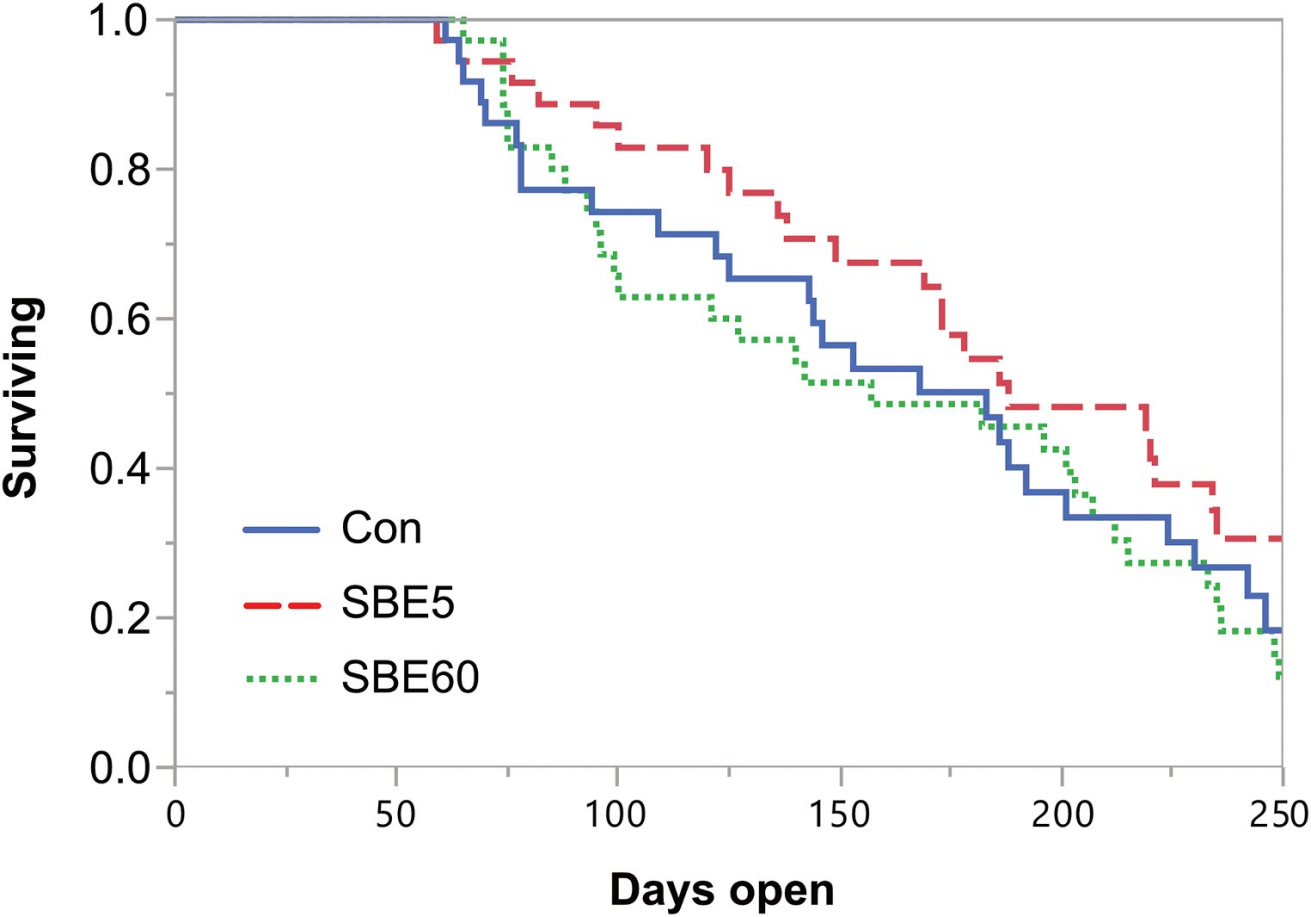
Survival plot for time to pregnancy through 250 DIM. Data were right-censored at 250 d. There was no treatment effect on pregnancy risk (*P* = 0.35). The hazard ratios for treatment comparisons were as follows: 0.71 (95% CI: 0.40–1.25) for SBE5 vs. Con and 1.13 (95% CI: 0.67–1.92) for SBE60 vs. Con.

**Fig 6:**
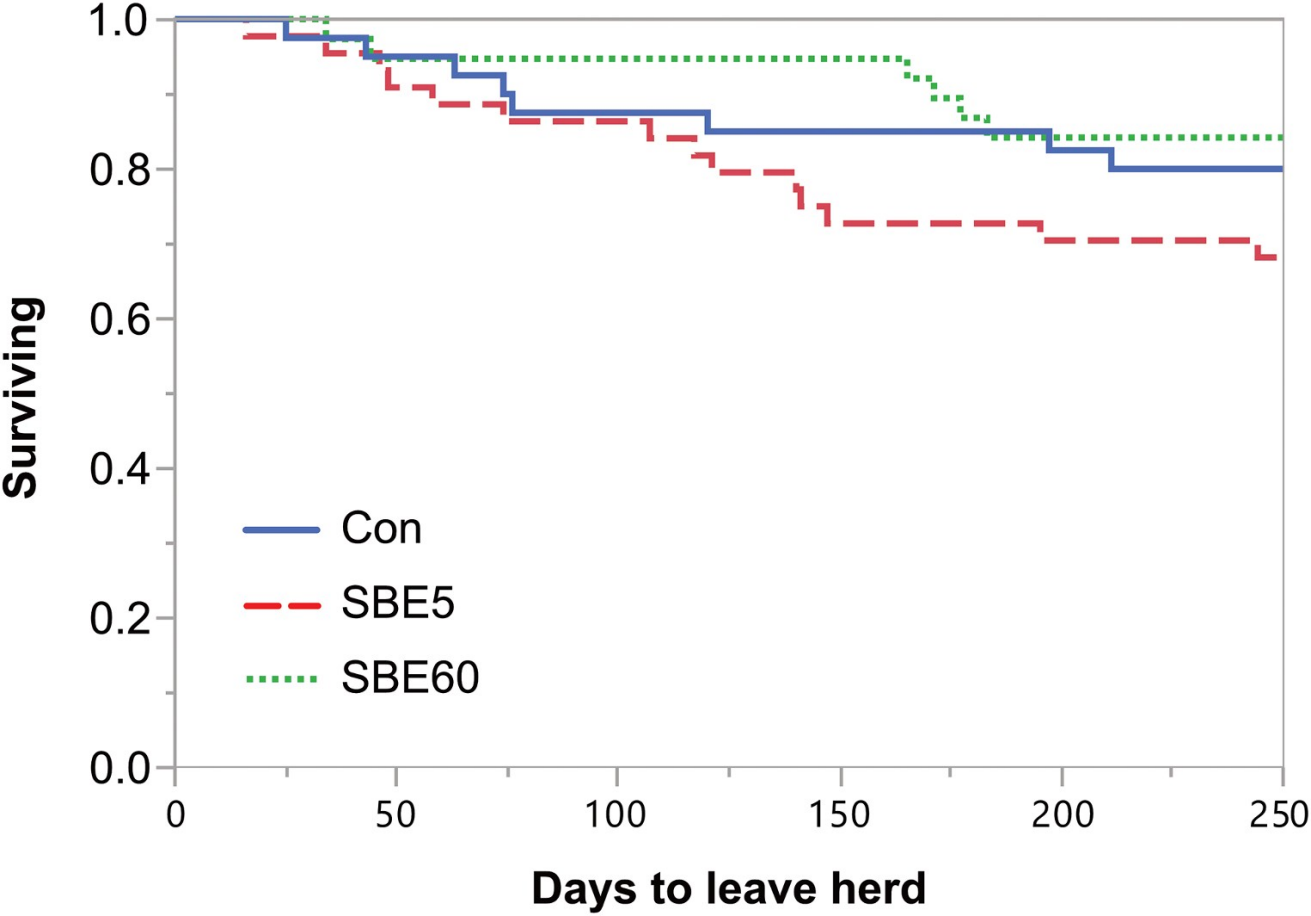
Surivival plot for time to leave the herd through 250 DIM. Data were right-censored at 250 d. The hazard of leaving the herd (cull or death) was not affected by treatment (*P* = 0.16). The hazard ratios for treatment comparisons were as follows: 1.85 (95% CI: 0.75–4.92) for SBE5 vs. Con and 0.72 (95% CI: 0.21–2.27) for SBE60 vs. Con.

Survival analyses through 250 DIM were completed for time to pregnancy (Fig 5) and removal from the herd (Fig 6). Treatment did not alter pregnancy risk (*P* = 0.35) and hazard ratios for treatment comparisons were 0.71 (95% CI: 0.40–1.25) for SBE5 vs. Con and 1.13 (95% CI: 0.67–1.92) for SBE60 vs. Con. Although herd removal risk did not differ by treatment (*P* = 0.16), cows with greater BCS at calving (≥ 4 vs. < 4) tended to have increased risk of removal (*P* = 0.06). The hazard ratios for herd removal were as follows: 1.85 (95% CI: 0.75–4.92) for SBE5 vs. Con and 0.72 (95% CI: 0.21–2.27) for SBE60 vs. Con.

## Discussion

The objective of the study was to determine the effects of *Scutellaria baicalensis* extract supplementation during early lactation on whole lactation milk production. The main flavonoids of SBE, baicalin, baicalein, wogonin, and wogonoside are known to have anti-inflammatory properties [28]. AMS adaptation during the first 2 DIM resulted in less than the target dose being provided, but from d 3 of lactation onward, the target mean dose of test material was successfully delivered by the AMS.

Scutellaria administration for 60 d effectively increased whole-lactation milk yield compared to control cows, whereas milk yield was not significantly altered in response to 5 d of supplementation. Treatment differences for SBE60 began at wk 4 of lactation, encompassed peak milk (wk 5), and progressed beyond the end of the treatment period, with nearly a 6 kg/d increase in peak milk compared to control (51.8, 50.0, 45.9 ± 1.96 kg/d). Similarly, in Tedesco et al. [16] cows fed the flavonoid silymarin from d 10 before expected calving to 15 DIM peaked 1 wk earlier and had increased peak yield (41.6 ± 1.05 vs. 39.1 ± 1.44 kg/d). Milk yield did not differ at 7 DIM, but was greater for silymarin cows at 21 and 30 DIM, as well as overall 305 d (9,922.1 ± 215.7 vs. 9,597.8 ± 225.4 kg; [16]). A slightly shortened length of silymarin and lycopene supplementation (7 d before expected calving to 14 DIM) also tended to increase milk yield during the first 21 d of lactation [29].

Longer duration of grape seed and grape marc meal extract (**GSGME**) supplementation from 3 wk prepartum to 9 wk after calving also increased milk yield during wk 4–6, with a tendency for greater yields at wk 3 and 7 of lactation [30]. Treatment for the same duration caused an 11% increase in ECM for cows supplemented with green tea and curcuma extract (**GTCE**) during wk 2–9 of lactation. When only investigated from 3 wk before to 3 wk after calving, intraduodenal administration of quercetin did not affect milk production [18]; however, given that milk yield responses to polyphenols have typically been seen at or after wk 3 of lactation, the shorter timeline of that study may have missed milk production responses occurring later in lactation [7].

Although previous polyphenol studies differed in duration of treatment administration, supplementation ranging from as little as 1 wk prior to expected calving to 14 d postpartum tended to increase milk yield in some studies. Even shorter postpartum treatment administration of NSAID (3 d) was effective at increasing whole lactation milk yield in previous work [7]. Despite the long-term impact of polyphenols or NSAID with treatment durations less than 60 d, milk yield response of SBE5 did not differ from control here. It is possible that 5 d administration of SBE increases milk production, but our observed response was insufficient to reach significance. The delay in milk response at or after 3 wk in lactation in our study and other flavonoid studies [16,17,30] suggests that these flavonoids may not simply be acting as an anti-inflammatory agents, but could also be having effects on DMI and ruminal microbial populations.

Considering the observation that total pellet allocation increased for SBE60 2 wk before milk yield differed, greater consumption of high energy concentrate feed could have contributed to the increased milk yield. Although total pellet allocation during the first 50 d of lactation was meant to be determined based on stage of lactation, we observed some deviation from these settings early in the study (see S1 File). After 50 DIM, feed allowance was based on milk yield, so after that point, increased milk yield of SBE60 cows triggered increased pellet allocation during the last 2 wk of the treatment period and throughout the carryover period. Although pellet provision was greater in SBE60 compared to SBE5 or control cows, the additional energy provided could not account for the magnitude of difference detected in milk yield. During d 1–63, SBE60 cows produced 4.46 kg/d more milk than control cows, which equates to 2.58 NE_L_ per day (0.69 Mcal/kg milk; [31]). The additional 0.42 kg pellet intake for SBE60 cows only supplies an additional 0.82 NE_L_/d. Although not measured in this study, it is likely SBE60 cows consumed more PMR. In previous polyphenol studies, the observed increases in milk yield were not matched by increases in DMI. Overall DMI during wk 2–9 was not influenced by periparturient feeding of GSGME (16.6 vs. 17.2 ± 0.63 kg/d; [30]) or GTCE (17.1 vs. 17.7 ± 0.59 kg/d; [17]). Additionally, DMI did not differ between treatments at any specific time point during wk 2–9 in either study.

Despite the greater energy demand that accompanies increased milk production, body condition score was not affected by treatment. Similarly, Tedesco et al. [16] observed no difference in prepartum BCS, but BCS losses from 15 d before calving to 30 DIM tended to be less for cows receiving silymarin. Other studies investigating polyphenol supplementation during the periparturient period did not report BCS [8, 17, 18]. We previously reported that NSAID treatment did not affect BCS despite inducing a 7–9% increase in whole-lactation milk yield [7]. The apparent lack of effect of anti-inflammatory agents on BCS in early lactation is most likely due to offsetting increases in DMI and milk yield, although improved digestive efficiency could play a role as well.

It is known that flavonoids are degraded by rumen microorganisms, the extent of degradation depends on flavonoid structure, and flavonoids alter ruminal ecology [32], but compared to monogastrics, there has been relatively little research on the mode of action of these flavonoids in ruminants. The few studies that have investigated the effects of flavonoids on ruminal parameters reported greater rumen pH [33], increased propionate production [33], and shifts in microbial populations [34]. It is possible SBE favorably altered rumen dynamics and diet digestibility, thereby increasing milk production in response to greater nutrient recovery from the diet.

Similar to the treatment differences observed for milk yield, milk lactose yield was greater in SBE60 compared to control, but not different between SBE5 and control. Timing of the observed differences in milk lactose yield coincided with increases in milk yield. Increased milk lactose synthesis could be a product of increased substrate available for lactose synthesis (glucose) or an increase in lactose synthesis pathway activity, either due to more mammary epithelial mass or greater activity per cell. Investigation of intraduodenal quercetin supplementation on glucose metabolism revealed no difference in hepatic mRNA abundance of enzymes involved in gluconeogenesis; however, no difference in lactose content was detected in that study [35]. Although those findings were in late lactation cows, previous studies supplementing polyphenols during the transition period also did not observe effects on milk lactose yield either [7, 9]. Similar to our observations, Garavaglia and others [29] observed a tendency for increased milk lactose yield (1.61 vs. 1.81 ± 0.07 kg/d) during the first 21 DIM for cows receiving silymarin and lycopene during the transition period.

Long term administration of Scutellaria also increased milk fat and protein yield. In previous transition cow studies, polyphenols either had no effect on milk protein yield [7, 17, 18] or increased milk protein by 9% [17]. Effects on milk fat yield tended to vary more in those studies, including a tendency for reduced milk fat content in cows receiving silymarin [16], no effect with GSGME supplementation [30], and 10 to 13% milk fat yield increases by GTCE [17] and silymarin and lycopene [29], respectively.

Increased mammary gland function indicated by increased lactogenesis and milk fat synthesis may be in response to improved mammary gland health. Both somatic cell count and incidence of mastitis were decreased in SBE60 with the timing of reduced SCC (wk 4–6) coinciding with increases in milk and milk lactose yields. Somatic cell count is indicative of mammary gland inflammation, which reduces synthetic capacity of the gland. Thus, SCC is negatively associated with milk yield [36] and yields of milk fat and lactose [37]. Similar to our results, Garavaglia and others [29] observed reduced early lactation SCC coinciding with increased milk fat yield and tendencies for increased milk yield and milk lactose yield. Although feeding a concentrated pomegranate extract (**CPE**) to early lactation cows did not significantly reduce SCC, milk yield increased by 6.4% [38]. Furthermore, Shabtay et al. [38] grouped mid-lactation cows into low SCC (**L-SCC**; <150,000 cells/mL milk) and high SCC cows (**H-SCC**; >150,000 cells/mL milk). L-SCC cows receiving CPE produced 1.9% more milk compared to their control and the proportion of CPE cows classified as H-SCC (>200,000 cells/mL milk) decreased by 22.8% compared to their control H-SSC counterparts. The reduction in milk and component yield during mammary gland inflammation can be at least partially attributed to the following factors: partitioning of glucose to support the increased presence of leukocytes, decreased uptake of nutrients by the mammary epithelium, and overall reduced synthetic capacity of the mammary epithelium [39]. Therefore, improved mammary gland health may provide a mechanism by which polyphenol supplementation increases milk production.

Milk haptoglobin concentration can be used to detect subclinical and clinical mastitis [40]; however, despite the observed decrease in SCC for SBE60, milk haptoglobin concentration did not differ by treatment. Given that milk samples analyzed for haptoglobin concentration did not extend beyond wk 2 of lactation and effects of SBE60 were not observed until wk 3 of lactation, it is likely SBE effects on mammary gland inflammation take longer to appear; however, this is based on only one biomarker.

Haptoglobin concentration in plasma is commonly used as a marker of systemic inflammation; however due to the ease of milk sampling with an AMS, milk haptoglobin concentration was used in this study to investigate mammary inflammation. Hiss and others [41] measured haptoglobin concentrations in both milk and serum samples. Haptoglobin concentrations were greatest in milk during wk 1 of lactation (54 ± 14 μg/mL) but decreased to basal levels by wk 6 (3.9 ± 0.8 μg/mL). Similarly, plasma concentrations were greatest during wk 1 (1600 ± 270 μg/mL) and decreased to basal levels by wk 3 (340 ± 90 μg/mL). Clearly, haptoglobin concentrations are greater in serum than milk, but the measures were correlated (r = 0.6). When Hiss et al. [42] induced mammary inflammation through intramammary injection of LPS, milk haptoglobin concentration increased relative to control quarters (152 ± 226 vs. 2.4 ± 1.1 μg/mL, respectively), but the magnitude of haptoglobin response was greater in blood (372 ± 38 μg/mL [42]. Although our d 1 milk haptoglobin concentrations were much less than those reported by Hiss et al. [41], values did decrease with DIM and reached a concentration lower than previously reported at wk 2.

Klein et al. [43] showed milk BHB concentration to be effective for identifying cows in metabolic stress and likely to develop ketosis. Previous studies have shown strong correlations between blood and milk BHB concentrations (r = 0.66 and 0.73; [44, 45]). Thus, even though a portion of BHB is utilized for de novo fatty acid synthesis in the mammary gland [46], milk BHB concentration remains reflective of plasma BHB concentrations. Milk BHB concentrations are typically less than plasma concentrations (69 ± 7 vs. 584 ± 27 μM, respectively), are increased in cases of clinical ketosis, and typically begin to increase by the second wk of lactation [30, 31].

Milk BHB concentrations in our study were greater than previously reported; however, BHB concentration did increase with DIM, similar to time effects observed in prior work [16]. *S*. *baicalensis* supplementation had no effect on milk BHB concentrations, which aligns with previous studies supplementing flavonoids and indicating no impacts on measures of hepatic ketogenesis [8–10]. Cows supplemented with GSGME did exhibit increased plasma BHB concentration during wk 1 to 5 of lactation compared to control [30]. Authors attributed the increase in BHB concentration to low glucose resulting from the need to support increased milk lactose yields in GSGME cows.

Energy balance is typically calculated using BCS, body weight, individual feed intake, and milk components. As several of these measures were impractical to obtain in this study, we used G6P in milk as an indirect proxy for energy balance. During early lactation, negative energy balance spurs lipid mobilization which induces oxidative stress. The mammary gland responds by increasing G6P concentration and shunting it to the pentose phosphate pathway for generation of NADPH [47]. Corresponding with this physiological shift in glucose utilization, milk G6P concentration is greatest during the first weeks of lactation [25] and Zachut et al. [47] have demonstrated a negative and linear correlation with energy balance (r = -0.45). Similar to previous studies [14, 33], milk G6P concentration did decrease with increased DIM in our study. The lack of treatment effect for milk G6P concentration, or G6P as a percent of milk glucose, suggests *Scutellaria* supplementation may not have influenced early lactation energy balance, consistent with the BCS results.

In a previous study, postpartum meloxicam administration tended to delay removal from the herd [7]. Although SBE supplementation induced milk responses similar to that caused by postpartum meloxicam treatment, supplementation of SBE for either 5 or 60 d did not alter herd retention. Increased milk yield alone, in the absence of negative effects on BCS and fertility, likely translates to economic and environmental benefits for the dairy industry. However, without data on the total dry matter intake of treatment groups, these putative benefits cannot be conclusively demonstrated.

The exact mechanism by which flavonoids improve ruminant productivity is not completely known, but decreased oxidative stress through decreases in reactive oxygen species is a potential mechanism reviewed by Gessner et al. [32]. Flavonoids in SBE decrease reactive oxygen species directly as a radical scavengers [9] and through upregulation of nuclear factor erythroid 2-related factor-2 [48], which induces transcription of anti-oxidative enzymes. Due to unknown extent of rumen degradation of flavonoids and the further biotransformation of flavonoids by detoxification pathways (methylation, glucuronidation, sulphation) in enterocytes and liver, many metabolites are produced that can be in circulation or reach the tissues [49]. Therefore, it is unknown to what extent effects in ruminants are due to the dietary flavonoids vs. their metabolites [32].

We aimed to determine if flavonoids from the SBE extract, baicalin, baicalein, and wogonin, would be transferred to milk; however they were not readily detectable by HPLC. Flavonoids derived from feed forages have been detected in milk [50], but we could not find other examples in which supplemental flavonoids were found in milk. Additionally, we believe this is the first study to analyze concentrations of these flavonoids in skim milk. Baicalin and baicalein have been detected in rat plasma by HPLC following intraveneous infusion of baicalein [51]. The flavonoid quercetin has been successfully measured in plasma of dairy cows after intra-ruminal application [14]. Our inability to detect the supplemented flavonoids in skim milk was likely due to a combination of the relatively low doses provided, incomplete bioavailability, potential transformation of the flavonoids, and potentially inefficient transfer to milk. Therefore, we conclude that provision of 100 g/d of the test material containing SBE was insufficient to deliver nutritionally meaningful quantities of these flavonoids to milk consumers.

## Conclusion

Supplementation of dairy cows with *Scutellaria baicalensis* extract for 60 d increased whole-lactation milk yield compared to control cows. Milk fat, protein, and lactose yields increased through 120 DIM and SCC was decreased during the treatment period for the 60-d treatment compared to control cows. Milk production parameters were not altered by short term administration (5-d) of SBE compared to control cows. Mastitis incidence was decreased by both short- and long-term administration of SBE, but no other impacts on health were observed, and time to pregnancy and herd retention were unaffected by treatment. Overall, 60-d administration of *S*. *baicalensis* extract increased milk production, but the unknown mechanisms underlying this response warrant further investigation.

## Supporting information

S1 File Analysis of pellet allocation by treatment and impacts on milk yield. (DOCX)
