# Supplementary material for: Dietary supplementation of Scutellaria baicalensis extract during early lactation decreases milk somatic cells and increases whole lactation milk yield in dairy cattle
Source: PLoS One. 2019 Jan 23;14(1):e0210744. doi: 10.1371/journal.pone.0210744 (PMC6343908; doi:10.1371/journal.pone.0210744)
Supplement: S1 File — (DOCX) [file pone.0210744.s001.docx]

**S1 Supporting Information**

In response to the unexpected increase in total pellet offered for SBE60 cows in wk 2 and 3
(S1 Fig), a more thorough investigation of the total pellet allocation was conducted. Pellet offered on an individual cow basis by day appeared to be increased for SBE60 cows, but only for cows calving prior to 4/6/16. Therefore, cows were clustered in 2 cohorts, those calving 2/23/16 to 4/5/16 (cohort 1) and those calving 4/6/16 to 5/23/16 (cohort 2). As shown in S1 Fig, cohort 1 cows on SBE60 received additional pelleted feed, particularly for cows being milked more frequently. In contrast, Con and SBE5 cows showed a steady increase in pellet offered across days, which did not depend on milking frequency, as the feed table was designed. In cohort 2, all treatments received the expected steady increase in pellet allocation, with no evident bias.


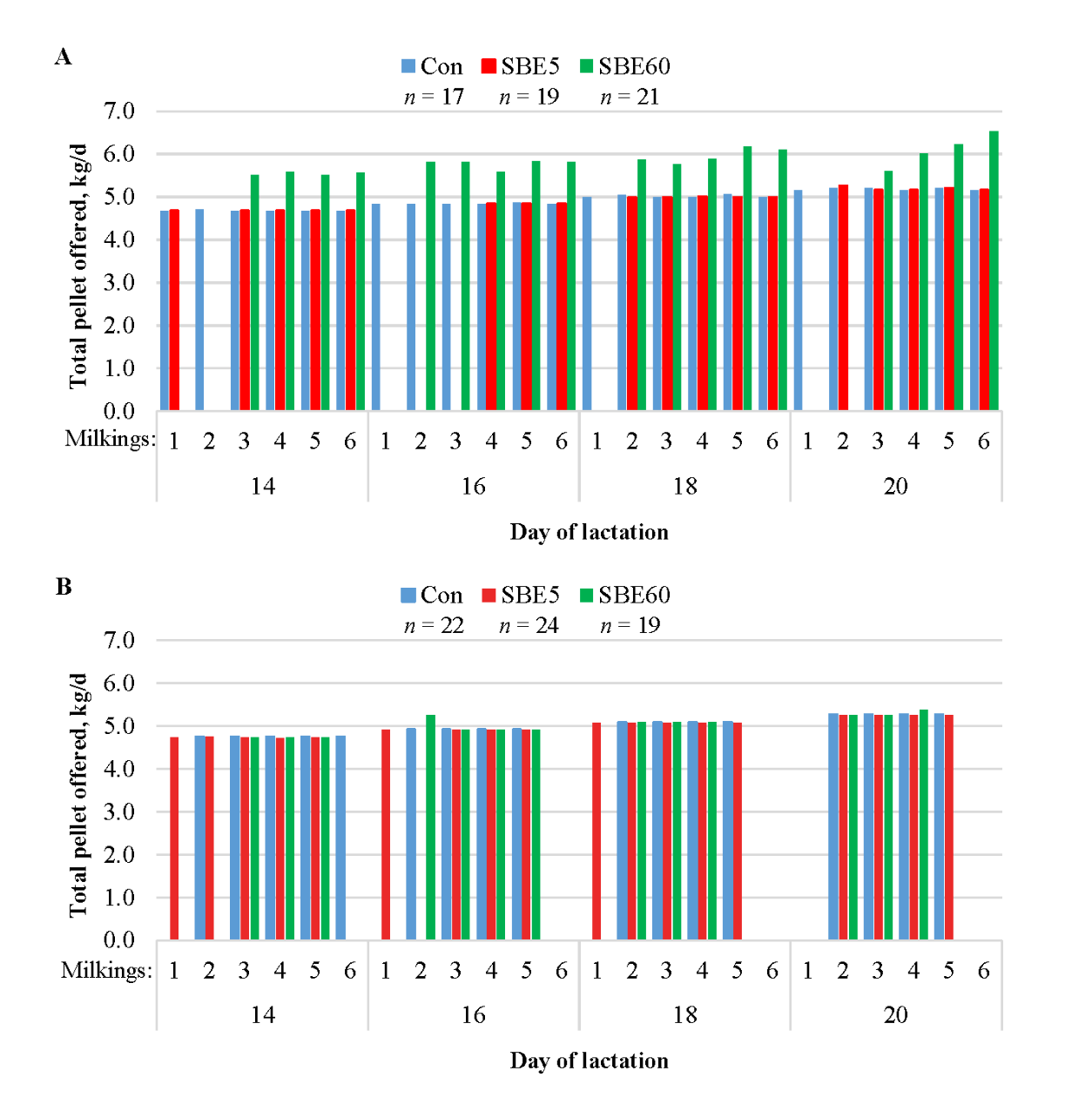
**S1 Fig. Total pellet offered from 17-21 DIM by milking frequency and treatment group for (A) cohort 1 and (B) cohort 2.** Mean pellet offered is shown for cows grouped by number of milkings for the day in question, by treatment.

Unfortunately, the T4C software (Lely) controlling this system does not log settings or changes to settings over time. However, the feed table employed for the study differed from the general herd feed table. It appears likely that the SBE60 group was erroneously assigned to the herd settings for feed adjustment during cohort 1, rather than the study setting; this only required a radio button toggle. Approximately half-way through the study, this setting was apparently corrected (cohort 2).

Considering the short-term differences in pellet allocation for SBE60, we were concerned about potential bias in dependent variables. Fortunately, the time-specific nature of the problem allowed us to empirically look for evidence of bias in our key outcomes. For whole-lactation milk yield, we re-assessed the data using the full model described in the main text but added cohort and cohort × treatment interaction terms to the model.

If the short-term increased allocation of pellet to SBE60 cows in cohort 1 explained the increase in whole-lactation milk yield, we would have expected a significant cohort × treatment interaction, reflecting an increase in milk yield for SBE60 only in cohort 1. However, there was no evidence of cohort or cohort × treatment effects in this analysis (S2 Fig). The model accounting for this unplanned treatment variable revealed a significant effect of SBE60 on whole-lactation milk yield, consistent with the results analyzed without a cohort effect. Thus, we are confident that treatment effects of SBE60 on 305-d milk yield are not primarily attributable to the altered feeding scheme for some cows on this treatment.

**S2 Fig. Whole-lactation (305-d) milk yields for treatments in cohorts 1 and 2.** Enrolled cows were divided into cohorts 1 (during the pellet allocation problem) and 2 (after the problem was resolved). Mean milk yield across 305 d was not influenced by cohort (*P* = 0.73) or cohort × treatment interaction (*P* = 0.62). *SBE60 differed from Con (*P* = 0.03), whereas SBE5 did not differ from Con (*P* = 0.54); treatment × week interaction was also significant (*P* = 0.02). Values are LS means ± SEM, *n* = 17 – 24.
